# Annual Meeting of the Monroe County Medical Society

**Published:** 1871-07

**Authors:** Chas. S. Starr

**Affiliations:** Secretary


ART. III.—*Annual Meeting of the Monroe County Medical Society.* June 14th, 1871.

———

Dr. H. W. DEAN, President, in the chair.

Dr. C. E. RIDER presented a patient who had been almost blind for a year, the effect of granular ophthalmia, both corneas having become almost wholly opaque. To relieve his condition, on the 9th of February last he made trial of innoculating the opacity with pus from the eyes of a child suffering from ophthalmia Neonatorum, and to-day the patient can readily count fingers at two feet distant, and the sight of both eyes is still steadily improving.

Dr. RIDER remarked that in cases where the encroaching blood-vessel cover two-thirds or more of the cornea, there is but little danger of corneal ulceration; and that in these very cases not amenable to other treatment, the innoculation of pus in some unknown manner clears away the pannus.

ANNUAL ADDRESS OF THE PRESIDENT, DR. H. W. DEAN.

Before entering upon the consideration of the special subject which I have proposed very briefly to occupy your time on this occasion, I wish to make a passing note of the working and potentia condition of our society. Comparing this with similar organizations in this State, the fact of our infrequent meeting requires explanation, which is this: So many of the members of this, are connected with the Rochester medical Society, it has been deemed not desirable that a more frequent than the annual meeting of the county should be attempted. Since my connection with this Society, now almost thirty years, of course a very material change has occurred in its membership. Then it was composed almost wholly of men past middle life, but a small number of them remain; but few young men then held membership here, and those few for the most part, constitute the senior members of this Sooiety. As no reflections of an invidious nature can arise from the comparison, I venture an allusion to another notably changed feature of our membership. A considerable number of the then members of this society, as also of the medical men in all this region of the State—meritorious and representative men in our profession—receive their rights to the priviliges and immunities of the profession through a license from the State or a County Medical Society. It seemed to me strange, then, that efforts at relaxation of statutory protection to the profession should be attempted. In common with many medical friends, I regretted the passage of the Act by our Legislature, in '44, by which all restraint upon practitioners of medicine was removed. But the observation of the working of this Act—for something more than a quarter of a century, has impressed me of the prudent forethought of the late Dr. Backus, then representing this senatorial district in our legislature, through whose influence largely, the Act became a law. It was designed by him to be the beginning of a series of efforts to place the profession of medicine beyond the contingencies of political legislation, to elevate its morals to a condition of independent self-protection. All subsequent legislation has been in the same direction. To-day we have, I think, evidence of the judiciousness of this policy, it is eminently consistent with the genius of our civil institutions, admitting of no exclusiveness in professional life, any more than in other business callings. A very large proportion of the additions to our profession, are young men who have had the advantages of critical intellectual culture preparatory to their medical course, and who, during their initiatory professional studies have had clinical advantages known to only a small proportion of medical pupils of a quarter a century ago. The advantages and successes of many of you, who have but just entered upon your professional career, mirror back to me the embarrassments under which I have labored, and always must labor. "Our glory is in our young men," and I bid you all God speed, assuring you that your highest aspirations in the pursuit of science, philosophy or art—also as benefactors of the race, will find abundant resources for their gratification in legitimate medicine.

Dr. Dean remarked that he had chosen his special subject, because of the murder trial, which had lately taken place here, and I was glad to see that Dr. Hammond, in his article, which appeared some weeks after his own was written, took similar views with himself on this subject:

RESPONSIBILITY OF EPILEPTICS.

Concerning what may be denominated the epileptic condition.

Proposition 1st—The literature of epilepsy, and facts obtained from most critical clinical observation, justify the following formula: That a susceptibility to epilepsy, after the period of early childhood, pre-supposes the existence of a peculiar permanent bodily condition, an individual idiosyncrasy of the nervous system, either congenital or acquired, without which, epileptic convulsions, or seizures would largely not be induced, and the existence of which would expose its possessor to epileptoid disease, on comparatively trifling provocation.

Prop. 2d.—All that is necessary to constitute an epileptic seizure "is a sudden and temporary, but absolute arrest of both perception and volition."

Prop. 3d.—A momentary aphasia, a single interruption of a step in walking, a sigh, are as characteristic of true epilepsy, as protracted suspension of consciousness, or chronic and long continued muscular convulsions.

Pathological changes, belonging to, or specifically characteristic of epilepsy, have not been determined. Patients known to have had epilepsy, dying from other disease, have exhibited no pathological change; in others, a mere trance of hyperæmia of the cerebral vascular tissue has been discovered. In others, again, cases of long standing, the medulla oblongata and its immediately elevated structure, as also, the peripheral parts of the brain, have been found in a condition of disorganization.

Epilepsy attended with chronic and long continued convulsions is most commonly followed with the *furor epilepticus*, an insanity of the senses rather than of the intellect, and ultimately resulting in that form of cerebral disorganization in which dementia, imbecility and idiocy are almost necessary consequences.

Concerning the petit mal of its influence on cerebration, testimony is abundant that the simple vertigenous seizures, or mild epileptic attacks, destroy the intellect more rapidly and more certainly than the more violent and protracted epileptic convulsions, and that patients demented from such causes are subject, under slight excitement, to maniacal outbursts, as with those subject to chronic muscular convulsion.

Accepting the general proposition relating to the epileptic condition, we accept a constitutional tendency to epilepsy—a special susceptibility to a specific disease. No argument is needed to convince the minds of medical men, that as a rule, epilepsy tends to the impairment of the functions of the brain. Exhibiting itself in the deterioration of mental power, especially in the exercise of attention, apprehension and ideation, excepting the wild fury—the furor epilepticus, which not unfrequently succeeds to chronic epileptic convulsions, there is wanting evidence of any physical condition consequent upon epilepsy, justifying the appellation of insanity. A very appreciable degree of dementia, the most frequent psycho-pathological sequel of epilepsy may exist, compatible with the power of correcting false impressions made through the sensuous organs upon the brain, hence in no degree diminishing individual responsibility. In addition to the mental impairment already mentioned, a popular interparoxysmal mental state of intensified passsions—irritability of extreme impressibility, often, though not as a rule, attaches to the epileptic condition. The literature of insanity—of insanity conditioned by epilepsy, is wanting in evidence of an instance of premeditative crime committed by an epileptic. Insanity complicating a criminal act committed by a subject of epilepsy, now, probably, will appear as an incident rather than as a consequence of epilepsy.

Conceding the epileptic bodily condition referred to, as also the interparoxysmal nervous and mental condition recognized in epileptics, except during the wild fury preceding or following a specific epileptic attack, the clinical history of epilepsy carefully scrutinized, affords but doubtful evidence at least, of incapacity on the part of its subjects to decide what acts are criminal and what are not.

Reports were received from standing committees, and under this head Dr. Kuichling read the following paper on

PAUPERISM.

Every state is constituted by the combination of all the families in a certain district of land, with the tendency of self-maintenance, preservation and mutual protection for the promotion of common wealth. Every body in the state obliged to contribute according to his ability to the promotion of the common wealth; so the rich by birth, the man prosperous by his own perseverance and activity, the common laborer, and even the poor, all contribute their share.

The activity of every member of the state is the basis of the common welfare. With the obligation of contributing is connected the right of mutual support in case of need. Whoever is by nature, merely as by a step-mother considered and endowed, or by misfortune, disease or old age, impoverished and crippled, and therefore unfit to acquire any longer by his own activity the absolutely necessary means of supporting his life, is a pauper, and has as such, the right *per se* to claim and to require the sympathy and assistance of his fellow citizens. The means of support are contributed by general taxation and voluntary donation and are distributed by the superintendent of the poor and his subordinates, the overseers of the towns. The support itself consists in presenting provisions, wood, light, money, medicine and medical assistance or in transferring the unfortunates to public institutions, alms houses, orphan asylums, asylums for the blind, the deaf and dumb hospitals, and hospitals for the insane.

The causes of pauperism, as far as they are within our reach, are chiefly neglected education, looseness of family morals, immortality, idleness, debauchery, misfortune, sickness, mutilation and old age; while the causes out of our reach are such as inundation, tempest, earthquake, drouth, stoppage of commerce, war, &c.; and to counteract these evils it will be our duty to take care for the education of children, especially by good schools; by improvement of manners and morals; by rousing the sense of honor; by temperate habits and industry.

In the country we find less poverty than in cities; in agricultural districts less than where there are many engaged in manufacturing; in the northern regions less than in the southern. So for example we find very few paupers in Sweden and Norway, both countries highly renowned for their sense of hospitality. With a refined manner of living and luxury goes pauperism always hand in hand. When Rome's star shone very bright poverty was very deep, settled and horrible. Expenses had to be incurred for the relief of the poor. We see the same thing in England. And if we direct our view to our own beloved country we find immense sums given away for the relief of the poor, increasing every year, not in proportion to the augmentation of the population, but keeping equal step with the advancing pomp, luxury, licentiousness and greediness of gain. And we shall not deceive ourselves by looking at these existing evils in our beloved county as having already assumed terrible dimensions; so that we have to regard them as threatening destroyers of our glorious republic. For with the neglect and decline of the true republican virtues, simplicity in manners and morals, and temperance, they will prove to be the pillars of despotism and monarchy.

In our country where there do not exist particular classes in society or distinctions acquired by birth, every one can work according to his choice; every laborer is esteemed for his work; no work is disgraceful. Whoever has the good will to do any work can find also the necessary means for supporting his life. It is therefore astonishing to see the number of poor increasing every year; and it is consequently our duty to be wise in distributing the means of support. Far be it from me to blame our officers in performing their duties. But I think it is not merely necessary to have established offices where the requisite support can be had in case of want; there should also be connected with their functions the duty of informing the poor where work is to be had. In this way the means for his support may easily be given to the applicant for alms, and at the same time the taxpayer can easily get the helping hand he needs. The good will to help the indigent is in intimate relation with the duty of improving the situation of the poor. Occupation is plenty. It is only necessary to know where it can be found. In maintaining this principle we do not need the especial societies of charity and benevolence, with which in general are combined particular sentimentality and a certain degree of vanity. It would be far better to put their crimes in the hands of the officers of the poor, as the relief can thus be made more uniform and extensive.

With these two methods of support there is also a third—namely, the establishment of colonies of the poor. They have especially been tried in Holland; but even in that comparatively small country this course has proved itself too costly, and is therefore a failure.

It may be said that many who have lived in better circumstances and lost their fortunes suddenly, without their own fault, will feel very bad to apply for support to the poor officer. This may be so. But I ask, is it not the same whose interference may be asked for, that of a friend or that of a respectable officer of the community. You, gentlemen, are health officers, and how many have called on you and asked your professional services! How many, I say, dressed in fine clothes, as well as clothed with rags, have received your costly advice—have occupied your time! How many operations have you performed on them! And still in these cases you have never received any compensation. You are in your social position on an equal footing with the other officers of the commonwealth; and all these persons did not feel ashamed to come to you for help.

Although it is the duty of everybody in the country to contribute to the common welfare his share according to his faculties; there are, nevertheless, a great many spending their time in idleness and still relying upon public charity. These must be admonished to do their duty, by forcing them to work; and for such the best place will be the workhouse.

Dr. E. M. Moore, from the Committee on epidemics and endemics reported that the city had been free, during the past year from any extensive or prevailing epidemic.

Dr. E. V. Stoddard read an article on

INFANT MORTALITY.

In reviewing the mortuary statistics of childhood in various countries, we are startled by the fact that such an enormous per cent of those born into the world die during the first five years of infant life. We propose briefly to consider the causes most active in the production of this condition, and the means for greatly lessening the evil. The mortality of children of all ranks in various countries below five years of age, varies from nineteen to fifty-five per cent. By far the greatest mortality occurring in the earlier months of the first year, and the per cent. of mortality in cities greatly exceeding that in the country. Amongst all classes of children the highest mortality is found in the foundling hospital. In the foundling hospital at Lyons and Parthenay the children are principally suckled, here we have a respective mortality of 33 and 35 per cent. In Paris, Rheims and Aix, artificial feeding is mainly resorted to, and we have a death-rate of 50, 63 and 80 per cent. In various institutions of our own, and other lands, where recourse is had to artificial feeding, we find a mortality ranging from 50 to 95 per cent. Considering these various statistics, it at first appears that a want of breast milk was the cause of the great difference; but however injurious this want may be, I cannot but think that there are other causes far more potent in causing this fearful sacrifice of human life. We must consider the crowding, insufficient ventilation, and especially the lack of proper care in the preparation of the infants food, necessarily existing in these institutions, as compared with the free air and the more careful and painstaking nursing of the young so universal in the country. In no nation of the world, I speak it with pride, is the value of individual human life so fully estimated as in the United States. It is to a peculiarity of our republican institutions that we owe this national tendency to rear and protect our young. With our uniformity of laws, free from the hampering prejudices of a State Church, not subject to the will or influence of individual authority, and gladly receiving any addition to our population—more than any other people, we are bound to care for the lives of each and all, and so far as possible to produce a healthy race of men and women. A recent German author states that 34 per cent. of the population of Europe is below fifteen years of age, and only 48 per cent between the ages of twenty and sixty, or the period of activity, *i. e.*, one half the population are consumers only. It is easy for us to see the necessity of prolonging life till a period is reached which shall have made the productive power of the individual equal to at least, if not greater than the amount of his consumption. Saving the life is not the whole of the duty incumbent; but infancy and youth, in their relations to the future, have a special claim upon us—that in so far as possible, we should reprove all causes which may interfere with the growth of a healthy, vigorous and well-developed mental and physical constitution. Congenital causes, such as malformations and any hereditary, feeble condition of the system, (in so far as this cannot be remedied,) are not to be reckoned in our estimate. One prominent cause of infant mortality, especially in cities, is zymotic disease. Any circumstances which produce a foul and vitiate atmosphere, are fatal to the well being of the infant. Another and the most important cause of mortality, is improper food and injudicious feeding. This cause I propose to consider especially. The food best adapted for the infant's wants is the mother's milk, and it should be regarded as the imperative duty of every mother to nurse her offspring. Whenever, however, it is necessaiy to find a substitute for this, it is for us to as nearly imitate the natural article as possible; and this we find to be the milk of some animal. Taking a number of analysis of different authorities, deducing therefrom an average, we conclude that next to human milk that most appropriate for the child is the milk of the cow, next of the goat, then that of the ass. The prominent constituents of milk—as water, casien, butter, &c., vary greatly in amount in the milk of different animals, and in the same animal at different times. The manner in which the casien of human milk and that of cow's milk curdles in the stomach, constitutes the distinguishing point between them. Casien of human milk curdles in light flakes, that of the cow in heavy lumps; and in substituting one for the other, a proper regard must be had for this varying action of the gastric juice on each. Again, cow's milk is more nearly neutral (sometimes even fatally acid) in its reaction than human milk. Remembering these main differences between the milk of the mother and that of the cow, we would call attention to a few simple, yet important points, to be remembered in the preparation and administration of artificial nutriment to children, care should be taken to secure milk from a cow which is properly fed and exercised. As cows milk, more easily than human, becomes acid, we must render it alkaline, by the addition of an alkaline solution. The best is a solution of bi-carbonate of soda in water, ʒj to ℥vi, of this a teaspoonful to be added to the milk at each feeding. Many conditions of the infants stomach exist, in which cows milk alone is not as well digested as when some farinaceous substance is mixed with it, as arrow root. These are usually cases of acid digestion, and here, the farinaceous food improves digestion in two ways: First, by adding to the milk its own nutritic properties, and more especially by retarding the coagulating of the casein, by minute duction, in such a manner that instead of coagulating into lumps, it forms small flakes, upon which the gastric fluid can act more easily and rapidly.

A nurture of cow's milk and wheat flour can be made, which contains all the blood making and caloric generating properties of human milk. Wheat flour, however, has an acid reaction, and needs the addition of an alkali. By its use, also, it adds another task to the digestive organs of the infant, viz: that of converting the starch of the wheat into sugar. We may accomplish this process before adding the flour to the child's food, by mixing malt meal with the flour. When milk and wheat flour are boiled together and malt meal is added to the gruel, while hot, the mixture assumes a sweet taste. This reaction having taken place and an alkali having been added to neutralize the acid of the flour, we have essentially Liebig's food. Liebig's formula is:

℞ Wheat Flour.
Malt Meal, aa ℥ ss.
Bi Carbonate Potassai, grs. vii ss.

Mix with the aid of water ℥j' afterwards adding milk ℥v. Heat upon a slow fire and stirring until it gradually becomes thick, remove it from the fire for a few minutes, stir, and again place upon the fire, remove it as before, again place on the fire and leave it till it boils. Remove the bran by straining. German authors highly praise this preparation as a substitute for the mother's milk.

I have said more upon the preparation of the infants food than the general character of this article would warrant, but I desired to present distinctly one or two points.

1st. That the present excessive mortality of infants is partly preventable.

2d. That the most prominent of the exciting causes is improper feeding in those reared artificially.

Thus, I think, by proper care, we may modify greatly, or entirely remove some of these causes. The choosing of the proper article of food is the primary consideration, and its proper preparation and administration in quantity and strength, is equally important. Here I must protest against the largely diluted food of infants. The custom of diluting the milk with an equal or even greater quantity of water is wrong. The amount of fluid has no effect on the digestability of the milk, onlp increases the work necessary for the stomach to absorb it. Ordinary cows milk does not contain twice the amount of blood-making and caloric generating material that is found in the human milk, and are we not, in following this custom, failing to imitate the natural food, and giving a less nourishing article of food than we design.

Absolute cleanliness is another essential requisite, the nursing bottle should be thoroughly washed in hot water after each feeding, and also in some alkaline solution to thoroughly cleanse it from any acidity.

The comparative mortality of cities and country remind us to insist on plenty of fresh air and sunshine.

Lastly, it is a mistake to feed infants with a spoon, food should always be given in a bottle with a properly constructed alkaline nipple. This necessitates the act of sucking, whereby the saliva is poured forth and thoroughly mingled with the food; which is very essential to the proper digestion of food in infants.

Dr. E. M. Moore asked if there were any facts to show if the difference of mortality in the foundling hospitals between those that used artificial feeding and those which employed wet nurses, could be accounted for by any other causes, *i. e.* by any difference in the ventilation or internal arrangements of these hospitals, more especially, he would suggest, it might be due to the less regular care those who were artificially fed received; and also that they, as a rule, obtained less fresh air and sunlight than those who feeding at the breast were therefore more commonly carried about by their nurses.

Dr. B. L. Hovey laid particular stress upon three points. 1st. The food should consist of the pure cows milk, and the top of it. 2d. That food should be given only at regular times and at stated intervals. 3d. That much of the domestic troubles of infants is due to too large an amount of food, whereby flatulence and colic are produced, the most perfect cure for this condition being oftentimes an emetic.

Dr. Chas. S. Starr, from the committee on obstetrics and diseases of women and children, read a report giving a *resume* of the year's literature on the latter branches. Special interest was shown to the varied views of the profession as to the propriety and use of intra-uterine medication, on which topic, though somewhat divided, the majority of testimony seems to favor its use. Also to the varied views as to the pathology and therapeutics of croup, especially to the use of the turpeth mineral, (see Dr. Barker's article) as an emetic in that disease. Amongst other items of this report we would call special attention to Dr. Byrd's Ready Method in Asphyxia (see Butler's half-yearly compend of Medical Science, January, 1871) to the fact that parenchymatous nephritis exists as a complication of other infantile diseases, in perhaps, one case in five, especially in cases of intestinal catarrh. (See Dr. Kjellberg's article in the same journal, half-yearly compend,) and to the interesting clinical observations of Dr. Fordyce Barker on malignant disease of the uterus, contained in the American Journal of obstetrics for March, 1871.

Dr. Llayton, of Spencerport, gave the detail of two cases of fractured clavicle, treated successfully and recovered from, without deformity, under the use of the bandage proposed to be used in this class of cases by Dr. Moore, at the meeting of the State Society at Albany in April, 1870. Dr. Montgomery also gave details of two cases treated according to this method, one of which proved a perfect success, the other a partial success. Er. E. M. Moore gave an account of an unique case of dislocation forward of the sternal end of the clavicle, which being reduced, and his bandage for fracture of the clavicle applied, was recovered from completely, and no deformity apparent.

Dr. B. L. Hovey, from the Committee on Surgery, read an account of a case of Traumatic Aneurism.

Dr. Hovey read a paper on Aneurism, and with it gave a detailed history of a case of Traumatic Aneurism, its cause, the pathology and increase of the disease, and his mode of treatment, with the result. The patient was forty years of age, good constitution, and a mechanic. The aneurism was situated in scarpal space, and caused by buckshot. The shot entered on the posterior side of the thigh, and was extracted immediately over the seat of the tumor. One year after the injury the aneurism was detected; the only treatment given for about five years was a tight compress over the tumor. This treatment did not prevent the increase of the disease. On or about the 1st of December last, the patient submitted to a course of treatment, which was continued, confining him for six weeks. The first process was to partially restrain the circulation, by compressing the artery as it passes over the pubic bone, this was continued twelve days. The integuments at this time were ulcerated, and the parts became so tender that it was deemed advisable to desist further treatment by this process. The second mode adopted was flexion. This was accomplished by confining the thigh to the pelvis, with a well adjusted pad securely fasted in the groin. This process was continued nine days. A third means was by making an instrument similar to a common truss, with a well adjusted pad applied over the artery as it passes over the pubis, and this pad forced upon the artery with a spiral spring. It is claimed by the author that by each of these means the same prinple of treatment was adopted, and that there is no other rational mode of imitating nature to produce a permanent cure of this disease.

The principle of treatment was to induce the formation of clot either in the supplying vessel or the aneurismal sac. This is done by moderating the flow of blood to the part.

When the blood is retarded in its normal force and quantity, through the sac, fibrine of blood is separated, and becomes a vital substance. This new formation unites to the coats of the sac, or new tissue already formed, and a cure is thus effected by occlusion of the sac or artery supplying it.

The result of the treatment in the case presented, was a success. The increasing and hard pulsating tumor had diminished is size one-half and was nearly solid and only a *slight thrill* was distinguished by the touch.

After the Annual Election of Officers and election of delegates to the State and American Medical Associations, the Society adjourned.

CHAS. S. STARR,

*Secretary.*

